# Beyond Spasticity: The Dual Impact of Selective Dorsal Rhizotomy in Spastic Quadriplegic Patients With Generalized Dystonia and the Need for Intrathecal Baclofen

**DOI:** 10.7759/cureus.83638

**Published:** 2025-05-07

**Authors:** Sevgi Sarikaya-Seiwert, Ralf Clauberg, Ina Hainmann, Hartmut Vatter, Hannes Haberl, Ehab Shabo

**Affiliations:** 1 Section of Pediatric Neurosurgery, Department of Neurosurgery, University Hospital Bonn, Bonn, DEU; 2 Department of Neuroradiology, University Hospital Bonn, Bonn, DEU; 3 Department of Pediatric Hematology and Oncology, University Hospital Bonn, Bonn, DEU; 4 Department of Neurosurgery, University Hospital Bonn, Bonn, DEU

**Keywords:** cerebral palsy, generalized dystonia, intrathecal baclofen therapy, motor function, quadriplegia, selective dorsal rhizotomy, spasticity

## Abstract

Background

Selective dorsal rhizotomy (SDR) is primarily indicated for spastic diplegia, effectively reducing lower limb spasticity. However, its role in generalized dystonia remains controversial, as some reports suggest potential symptom exacerbation. In such cases, intrathecal baclofen (ITB) represents the preferred approach. This study evaluates the effects of SDR as a secondary intervention following insufficient ITB therapy on motor function in patients with spastic quadriplegic cerebral palsy (CP) and generalized dystonia while also introducing a novel surgical strategy to approach mixed movement disorders.

Material and methods

This retrospective study included patients with spastic quadriplegic cerebral palsy and generalized dystonia treated at our institution between 2018 and 2023. All patients (n = 16) initially received intrathecal baclofen (ITB) therapy. In three patients, ITB monotherapy was effective in symptom control; however, due to insufficient spasticity management in the remaining 13 patients, selective dorsal rhizotomy (SDR) was subsequently performed without removal of the existing ITB system. Due to the worsening of dystonia and the lack of significant improvement in motor functions after SDR, ITB therapy was reintroduced. Clinical outcomes, including Gross Motor Function Classification System (GMFCS), Modified Ashworth Scale (MAS), and dystonia severity, were assessed before and after SDR, as well as following ITB reinitiation. Additionally, alterations in required ITB dosage before and after SDR were analyzed.

Results

While SDR effectively reduced spasticity in all patients, no improvement in overall motor function was observed. Notably, 69.2% of patients showed worsening of dystonia after SDR. ITB therapy was reinitiated in 11 patients (84.6%). Subsequent clinical evaluation revealed a significant improvement in both dystonia and all motor functions (standing, sitting, and transitional movements) across all patients (p<0.001). Furthermore, the required dosage of ITB to control dystonia after SDR was significantly lower than the required dosage before SDR (p<0.001).

Conclusion

Our findings suggest that a tailored, multimodal approach is essential for managing complex cases of CP with spasticity and dystonia. Furthermore, retaining the ITB system without explantation when performing SDR may be a viable strategy that could reduce the overall surgical burden and associated risks for the patient.

## Introduction

Cerebral palsy (CP) is a group of permanent movement disorders that emerge in early childhood, primarily affecting movement and posture. CP is classified into four major motor types: spastic, dyskinetic, ataxic, and mixed [1,2], with spastic CP being the most prevalent, affecting approximately 80%-95% of diagnosed individuals [1,3].

Spasticity, characterized by increased muscle tone leading to stiffness and restricted movement, significantly impacts mobility, daily activities, and overall quality of life [1-3]. Many patients diagnosed with spastic CP also exhibit various degrees of dystonia, a movement disorder marked by involuntary muscle contractions that cause repetitive movements or abnormal postures [1,2].

Given the complexity of these motor impairments, accurate assessment tools, such as the modified Ashworth scale (MAS) for muscle tone evaluation and the Gross Motor Function Classification System (GMFCS) for functional classification, are crucial in clinical decision-making [4,5].

Among non-ambulatory children with severe spasticity or mixed CP, surgical interventions such as selective dorsal rhizotomy (SDR) and intrathecal baclofen (ITB) therapy are commonly utilized to manage symptoms [6-9].

ITB is typically preferred for patients with generalized spasticity or mixed movement disorders, as it effectively reduces spasticity in both the upper and lower extremities while also alleviating dystonia-related pain [2,6,7,10-12]. SDR, on the other hand, is primarily indicated for ambulatory children with spastic diplegia, where the goal is to improve gait and overall mobility [2,11,13-15].

Numerous studies have demonstrated SDR’s efficacy in reducing lower limb spasticity [3,16,17]. Its role in managing accompanied generalized dystonia remains controversial. Some reports suggest that SDR may exacerbate dystonic symptoms in certain patients, highlighting the importance of careful patient selection and individualized treatment planning [18].

Despite extensive research on ITB and SDR separately, there is a notable lack of studies analyzing functional neurological outcomes in complex cases involving both spasticity and generalized dystonia. Moreover, there is limited guidance on optimal surgical strategies when ITB therapy fails. In such cases, one potential approach involves the removal of the ITB pump system followed by SDR [2,19,20].

This study aims to evaluate the efficacy of SDR as a secondary intervention in conjunction with the reinitiation of ITB therapy in patients with complex movement disorders, specifically those diagnosed with spastic quadriplegic CP and concurrent generalized dystonia. Additionally, we propose a novel surgical strategy for managing patients exhibiting mixed movement disorders characterized by the coexistence of spasticity and generalized dystonia. This strategy seeks to optimize functional outcomes, minimize potential surgical complications, and enhance overall quality of life.

## Materials and methods

Patients’ selection and inclusion criteria

Between 2018 and 2023, all patients (n = 16) with spastic quadriplegic cerebral palsy and generalized dystonia initially received intrathecal baclofen (ITB) therapy at our institution. ITB monotherapy was effective in only three patients; in the remaining 13, spasticity control was insufficient, necessitating subsequent selective dorsal rhizotomy (SDR) without removal of the existing ITB system. Accordingly, this retrospective study includes all patients (n = 13) who underwent SDR following inadequate response to ITB monotherapy.

Surgical approach

Initially, all patients underwent intrathecal baclofen (ITB) therapy due to concurrent generalized dystonia. However, over time, these patients exhibited suboptimal spasticity control with ITB monotherapy. As a result, selective dorsal rhizotomy (SDR) was conducted.

Once the patients were approved for SDR, ITB therapy was gradually weaned in preparation for the procedure. They did not receive intrathecal baclofen for at least one month before SDR to prevent baclofen withdrawal symptoms such as increased spasticity (rebound effect), agitation, hallucinations, seizures, and, in severe cases, delirium or multi-organ failure.

SDR is performed under general anesthesia with the patient in the prone position, using standard microsurgical techniques. The procedure involves a single-level laminotomy, most commonly at the L1-L2 level, as determined preoperatively by spinal MRI to accurately localize the conus medullaris. Intraoperative electrophysiological stimulation is then employed to identify hyperactive dorsal (sensory) rootlets, which are selectively sectioned. Based on stimulation response, approximately 60-80% of the abnormal rootlets are bilaterally divided to achieve optimal spasticity reduction while preserving motor function.

Given the challenges associated with the prone positioning required for SDR and the additional risks of prolonged operative time and complications related to intraoperative repositioning to side position for explantation of the ITB system, the ITB system was not explanted after completing SDR.

Multidimensional assessment of movement disorders was conducted before and after the SDR procedure using the following scales: Gross Motor Function Classification System (GMFCS), a widely used, standardized, and five-level classification system that classifies motor function in children with cerebral palsy based on self-initiated movement, with level I indicating minimal motor function impairment, whereas level V represents severe limitations in voluntary movement, requiring extensive use of assistive technology [5]. For spasticity, we used the Modified Ashworth Scale (MAS), a clinical tool that assesses the resistance during passive muscle stretching and ranges from 0 (no increase in muscle tone) to 4 (affected part(s) rigid in flexion or extension), providing a semi-quantitative measure of hypertonia [21]. Dystonia was assessed mainly based on clinical observation of involuntary muscle contractions that result in repetitive movements and abnormal postures; however, the Fahn-Marsden Dystonia Rating Scale (FMDRS) [22], composed of two clinician-rated subscales (a movement subscale ranging from 0 to 120 points, based on patient examination, and a disability subscale ranging from 0 to 30 points, based on the patient’s report of disability in activities of daily living), was used as a supportive tool and not as a primary tool due to its limitations, such as the absence of detailed assessment of individual body areas, such as separate ratings for proximal and distal limbs [22].

Specific functional abilities were evaluated using a two-step approach. First, a visual gait assessment scale was applied [23], followed by a clinical evaluation of three key motor function skills: standing, sitting, and transition movements. Functional outcomes were categorized as “improved,” “unchanged,” or “worsened” based this dual approach.

If the dystonia and the motor functions (standing, sitting and transition movement) did not improve after SDR during the initial postoperative evaluation within four weeks, reinitiation of ITB therapy was indicated. Due to the remaining ITB system, no further surgery was needed to reinitiate ITB therapy.

Following the combined approach (SDR and ITB) as well as during follow-ups, dystonia and motor functions (standing, sitting and transition movement) were thoroughly assessed using the same standardized approach of presurgical evaluation using GMFCS, AMS and visual gait assessment scale.

The clinical evaluation of movement disorders in all patients was conducted, at the time of first diagnosis as well as during follow-ups, in an interdisciplinary setting involving neurosurgeons, orthopedic surgeons, neuro-pediatricians, and physiotherapists.

The required dosage of intrathecal baclofen to effectively reduce the dystonia without causing any side effects before and after SDR in µg/day was also analyzed.

Statistical analysis

All data analysis was performed using IBM Corp. Released 2020. IBM SPSS Statistics for Windows, Version 27.0. Armonk, NY: IBM Corp. In the case of categorical variables, data are given as numbers and percentages. After normality testing via the Shapiro-Wilk test, continuous normally distributed data were compared using t-tests, while the Mann-Whitney U test was used for non-parametric data. Nominal data were tested between groups using Fisher’s exact test and in the case of multinomial data with a chi-squared test.

A p-value of <0.05 was considered statistically significant.

## Results

Patient cohort and general characteristics

A total of 13 patients (nine males, four females) with generalized dystonia and severe spastic quadriplegic cerebral palsy (CP) were included in this study. The average age at CP diagnosis was 20.2 months (range: 12-31 months).

Due to the absence of improvement in spasticity and motor impairments, all patients underwent a secondary SDR. Patients had an average of 4.8 years (range: 3-8 years) of intrathecal ITB therapy before undergoing SDR. The average age at SDR was 12.3 years (range: 9-16 years). The mean follow-up time after SDR was 11.2 months (range: 6-16 months). Patients’ characteristics are summarized in Table 1.

**Table 1:** Demonstrates patients characteristics prior to SDR. GMFCS: Gross Motor Function Classification System; ITB: Intrathecal Baclofen Therapy; MAS: Modified Ashworth Scale; No.: Number; SDR: Selective Dorsal Rhizotomy

No. of Patients		13
Age at Diagnosis (months)		20.2 (range: 12-31)
Male/Female		9/4
GMFCS	Level IV	1
	Level V	12
MAS score	3	2
	4	11
Time of ITB (years)		4.8 years (range: 3-8 years)
Age at SDR		12.3 years (range: 9-16 years)
Follow-up time after SDR		11.2 months (range: 6-16 months)

Development of spasticity, motor function, and dystonia after SDR

GMFCS level, MAS score, and specific functional abilities developed after SDR as follows:

GMFCS level: Before and after SDR, all patients remained at the highest level of impairment (GMFCS level V) with one patient even worsening from GMFCS level IV pre-SDR to GMFCS level V post-SDR. This indicates that SDR did not significantly improve overall mobility.

MAS scores: Before SDR, 11 had a MAS score of 4 and two patients had an MAS score of 3. After SDR, this score dropped to 0 in all patients, indicating a successful reduction of spasticity.

Standing: Eight patients (61.5%) showed no change, while five patients (38.5%) improved.

Sitting: Four patients (30.8%) showed no change, while nine patients (69.2%) worsened.

Transition movements: All patients (100%) exhibited no changes in transition movements.

Dystonia: 9 out of 13 patients (69.2%) experienced worsening of dystonia after SDR. The rest four patients (30.8%) had stable dystonia.

Development of motor function and dystonia after ITB reinitiation

Since most of the patients experienced worsening dystonia and none of the patients had an improvement of overall motor function after the SDR surgery, oral baclofen therapy was started in patient number 1 three weeks after SDR. The symptoms got slightly better with a baclofen dose of 30 mg/day. Due to this notable improvement, intrathecal ITB therapy was reintroduced to control dystonia in all patients. Two families (patients 7 and 11) opted against renewed ITB therapy. In one case (patient 1), a new central ITB catheter was implanted due to a previously removed central catheter during the SDR surgery.

Overall, 11 of 13 patients (84.6%) underwent a reinitiation of ITB therapy. Remarkably, all 11 patients (100%) who underwent reinitiation of ITB therapy exhibited a significant improvement in dystonia and all functional abilities (standing, sitting, and transition movements) with a p-value of <0.001.

Furthermore, the required dosage of intrathecal baclofen to control dystonia after SDR (175±27.4µg/d) was significantly lower than the required dosage before SDR (485±61.5µg/d) with a p-value of <0.001 in both t-test and Mann-Whitney U test.

A detailed comparison of motor function and dystonia progression following SDR alone and after the combined approach (SDR and ITB) as well as the required ITB dosage is presented in Table 2.

**Table 2:** Statistically compares the progress of motor functions and dystonia after SDR alone and after initiation of ITB following SDR. ITB: Intrathecal baclofen therapy; µg/d: Microgram per day; No.: Number; SD: Standard deviation; SDR: Selective dorsal rhizotomy

		SDR alone	ITB after SDR	p-values
Patients No. (%)		13	11	
Spasticity	improved	13 (100%)	remained improved after SDR	p = 1.0
	no change	0		
	worsened	0		
Dystonia	improved	0	11 (100%)	p < 0.001
	no change	4 (30.8%)	0	
	worsened	9 (69.2%)	0	
Standing	improved	5 (38.5%)	11 (100%)	p < 0.001
	no change	8 (61.5%)	0	
	worsened	0	0	
Sitting	improved	0	11 (100%)	p < 0.001
	no change	4 (30.8%)	0	
	worsened	9 (69.2%)	0	
Transition movements	improved	0	11 (100%)	p < 0.001
	no change	13 (100%)	0	
	worsened	0	0	
Required dosage of intrathecal Baclofen (Median ± SD) in µg/d		485±61.5	175±27.4	p < 0.001

Details about patients’ characteristics and motor function development are presented in Table 3.

**Table 3:** Detailed patients' characteristics. CP: Cerebral palsy; GMFCS: Gross motor function classification system; ITB: Intrathecal baclofen therapy; MAS: Modified Ashworth Scale; µg/d: Microgram per day; N.D.: Not determined; SDR: Selective dorsal rhizotomy

Patient Number	Age at Diagnosis (months)	Years with ITB	ITB Dosage pre-SDR (µg/d)	Age at SDR (years)	ITB Removal	Follow-up (months)	Pre-SDR GMFCS	Post-SDR GMFCS	Pre-SDR MAS	Post-SDR MAS	Dystonia after SDR	Post-SDR Standing	Post-SDR Sitting	Post-SDR Transition Movement	Re-ITB Therapy	Dystonia (SDR+ITB)	Standing (SDR + ITB)	Sitting (SDR + ITB)	Transition Movement (SDR + ITB)	ITB Dosage post-SDR (µg/d)
1	18	4	535	9	Yes	16	5	5	4	0	worsened	no change	worsened	no change	yes	improved	improved	improved	improved	170
2	15	6	512	12	No	14	5	5	4	0	worsened	no change	worsened	no change	yes	improved	improved	improved	improved	175
3	20	4	487	13	No	14	5	5	4	0	worsened	no change	worsened	no change	yes	improved	improved	improved	improved	235
4	24	3	420	14	No	13	5	5	3	0	no change	improved	no change	no change	yes	improved	improved	improved	improved	205
5	17	5	387	14	No	11	4	5	3	0	no change	improved	no change	no change	yes	improved	improved	improved	improved	175
6	12	4	400	13	No	11	5	5	4	0	worsened	no change	worsened	no change	yes	improved	improved	improved	improved	150
7	25	5	575	15	Yes	10	5	5	4	0	no change	no change	no change	no change	no	n. d.	n. d.	n. d.	n. d.	0
8	30	7	455	15	No	10	5	5	4	0	worsened	improved	worsened	no change	yes	improved	improved	improved	improved	210
9	27	8	415	16	No	8	5	5	4	0	worsened	no change	worsened	no change	yes	improved	improved	improved	improved	150
10	31	4	485	12	No	8	5	5	4	0	worsened	improved	no change	no change	yes	improved	improved	improved	improved	155
11	22	4	550	11	Yes	7	5	5	4	0	worsened	improved	worsened	no change	no	n. d.	n. d.	n. d.	n. d.	0
12	19	3	420	11	No	6	5	5	4	0	no change	no change	worsened	no change	yes	improved	improved	improved	improved	175
13	18	4	510	10	No	6	5	5	4	0	worsened	no change	worsened	no change	yes	improved	improved	improved	improved	200

**Figure 1:**
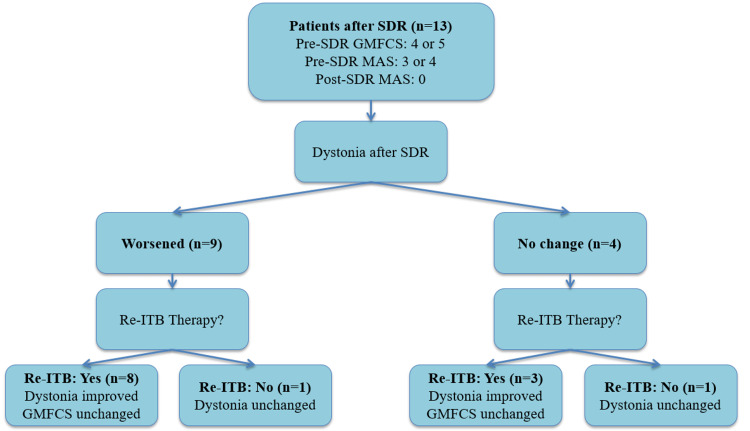
Illustrates motor function outcomes (GMFCS level, MAS score and dystonia status) following the reinitiation of intrathecal baclofen (ITB) therapy after selective dorsal rhizotomy (SDR).

To facilitate better comprehension of the detailed tables presented above, the results are summarized in an easily interpretable visual format in Figure 1.

## Discussion

Selective dorsal rhizotomy (SDR) is well-established for managing spasticity, but its effectiveness in cases with concurrent generalized dystonia remains uncertain, with some studies suggesting it may worsen dystonic symptoms [18]. This underscores the importance of careful patient selection, with ITB therapy typically being the preferred approach for generalized dystonia. However, there is a lack of research on the functional outcomes of treating complex and mixed cases with both spasticity and generalized dystonia and no reports on the combined or simultaneous use of the two approaches (SDR and traumatic brain injury [TBI]) [24].

Our retrospective study examines the impact of SDR as a secondary intervention following insufficient initial ITB therapy, then combined with ITB reinitiation in patients with spastic quadriplegic CP and coexisting generalized dystonia, proposing a novel surgical approach for these mixed movement disorders.

Limited studies in the literature have investigated the effectiveness of secondary SDR following unsuccessful ITB therapy for spasticity management [2,19,20]. These studies describe a procedure in which, after completing SDR, patients are repositioned for baclofen system removal with separate incisions made on the back and abdomen to extract the baclofen pump and catheters [2,20,24].

This additional surgical step logically and inevitably extends the overall operative duration, potentially increasing the risk of surgical complications. However, information about extended surgery duration and postoperative complications was not reported in the existing literature. In our institute, we followed this in our first case routine by removing the intrathecal part of the catheter of the baclofen pump. We disconnected the system from the connection of the intrathecal catheter with the pump. To avoid potential risks associated with intraoperative repositioning of the patient and prolonged surgery, we decided, to retain the baclofen pump after completing the SDR. The surgery for removal of the pump was scheduled three months after the SDR surgery.

Consistent with previous findings in the literature, we observed that SDR effectively reduced spasticity, as demonstrated by a decrease in MAS scores from 4 to 0 in all patients. However, it did not lead to improvements in gross motor function, with GMFCS levels remaining at level V. Moreover, SDR alone was insufficient in managing dystonia, as 69.2% of cases experienced a worsening of symptoms, requiring the reintroduction of ITB therapy. Notably, patients who received ITB reinitiation following SDR showed functional improvements in 100% of cases, including enhanced abilities to sit, stand, and perform transition movements. These results suggest that the reintroduction of ITB therapy after SDR significantly improves functional outcomes in patients with mixed severe quadriplegic spastic CP and generalized dystonia.

These findings underscore the limitations of SDR in cases where dystonia coexists with spasticity. While SDR effectively reduces spasticity, its potential to exacerbate dystonic symptoms highlights the need for individualized treatment strategies.

The success of ITB reinitiation after SDR suggests that a combined approach may be beneficial for selected patients, reinforcing the importance of thorough preoperative assessment and personalized treatment planning.

Furthermore, our study suggests that in patients with mixed motor function impairments who have experienced an inadequate response to ITB therapy, performing SDR without explanting the existing ITB system during the SDR procedure may represent a safe and effective management strategy.

This strategy could minimize potential surgical complications as previously mentioned as well as eliminate the need for an additional surgical procedure in case ITB therapy should be reinstated, thereby reducing surgical burden and associated risks for the patient.

Overall, this study represents the first report in the literature addressing this highly complex subgroup of CP patients, characterized by quadriplegic involvement and generalized dystonia. Additionally, it introduces a novel surgical approach that has not been previously described. This technique is designed to minimize potential surgical complications by reducing operative time while also improving the clinical outcomes of these patients.

The present study is, however, limited by its retrospective design, small patient cohort, and single-center setting, which may restrict the statistical power and affect the external validity and generalizability of the results.

Larger, multicenter prospective studies are needed to validate the findings of this study and, if proven beneficial, standardize the dual surgical approach in highly complex CP cases.

## Conclusions

Selective dorsal rhizotomy remains a valuable surgical option for reducing spasticity but does not enhance motor function in quadriplegic patients with concurrent generalized dystonia. Moreover, it may contribute to worsening dystonia in the majority of cases, underscoring the need for ITB reinitiation after SDR. These findings highlight the importance of a carefully tailored, multimodal approach to complex and mixed cerebral palsy cases with spasticity and dystonia.
